# Harnessing native corticosteroid-binding globulin to treat life-threatening septic shock

**DOI:** 10.1210/endocr/bqag002

**Published:** 2026-01-12

**Authors:** Stewart D Ramsay, Declan E Kilgariff, Benjamin J Young, Luke C Darveniza, Ryan L O’Hare Doig, Courtney A Hollis, Plinio R Hurtado, Mark P Plummer, Edward G Robins, Jessica H Lee, Emily J Meyer, Marni A Nenke, David J Torpy, Richard L Young

**Affiliations:** Intestinal Sensing Group, University of Adelaide, Adelaide, SA 5005, Australia; Adelaide Medical School, University of Adelaide, Adelaide, SA 5005, Australia; Lifelong Health, South Australian Health & Medical Research Institute (SAHMRI), Adelaide, SA 5000, Australia; Intestinal Sensing Group, University of Adelaide, Adelaide, SA 5005, Australia; Adelaide Medical School, University of Adelaide, Adelaide, SA 5005, Australia; Intestinal Sensing Group, University of Adelaide, Adelaide, SA 5005, Australia; Adelaide Medical School, University of Adelaide, Adelaide, SA 5005, Australia; Intensive Care Unit, Royal Adelaide Hospital, Adelaide, SA 5000, Australia; Molecular Imaging and Therapy Research Unit, South Australian Health & Medical Research Institute (SAHMRI), Adelaide, SA 5000, Australia; Adelaide Medical School, University of Adelaide, Adelaide, SA 5005, Australia; Molecular Imaging and Therapy Research Unit, South Australian Health & Medical Research Institute (SAHMRI), Adelaide, SA 5000, Australia; Molecular Imaging and Therapy Research Unit, South Australian Health & Medical Research Institute (SAHMRI), Adelaide, SA 5000, Australia; Adelaide Medical School, University of Adelaide, Adelaide, SA 5005, Australia; Adelaide Medical School, University of Adelaide, Adelaide, SA 5005, Australia; Intensive Care Unit, Royal Adelaide Hospital, Adelaide, SA 5000, Australia; Lifelong Health, South Australian Health & Medical Research Institute (SAHMRI), Adelaide, SA 5000, Australia; Molecular Imaging and Therapy Research Unit, South Australian Health & Medical Research Institute (SAHMRI), Adelaide, SA 5000, Australia; Adelaide Medical School, University of Adelaide, Adelaide, SA 5005, Australia; Endocrine and Metabolic Unit, Royal Adelaide Hospital, Adelaide, SA 5000, Australia; Adelaide Medical School, University of Adelaide, Adelaide, SA 5005, Australia; Endocrine and Metabolic Unit, Royal Adelaide Hospital, Adelaide, SA 5000, Australia; Endocrine and Diabetes Services, Queen Elizabeth Hospital, Woodville South, SA 5011, Australia; Adelaide Medical School, University of Adelaide, Adelaide, SA 5005, Australia; Endocrine and Metabolic Unit, Royal Adelaide Hospital, Adelaide, SA 5000, Australia; Adelaide Medical School, University of Adelaide, Adelaide, SA 5005, Australia; Endocrine and Metabolic Unit, Royal Adelaide Hospital, Adelaide, SA 5000, Australia; Intestinal Sensing Group, University of Adelaide, Adelaide, SA 5005, Australia; Adelaide Medical School, University of Adelaide, Adelaide, SA 5005, Australia; Lifelong Health, South Australian Health & Medical Research Institute (SAHMRI), Adelaide, SA 5000, Australia; Research Centre Translating Nutritional Science to Good Health, University of Adelaide, Adelaide, SA 5005, Australia

**Keywords:** sepsis, septic shock, corticosteroid-binding globulin, inflammation

## Abstract

Septic shock urgently requires new treatments. We reported that low circulating concentrations of the native glucocorticoid carrier, corticosteroid-binding globulin (CBG), predict a 3-fold increase in human septic shock mortality. To explore this, we used our murine model of high-grade polymicrobial sepsis (cecal ligation and puncture [CLP]) to test CBG therapy.

We prefitted adult male C57BL/6 mice (n = 106) with wireless arterial telemetry, then induced high-grade CLP. Mice were randomized with or without intravenous CBG therapy at 6 hours (3.5 mg/kg) and 30 hours (2.5 mg/kg). Terminal bloods, collected on humane endpoints or at 96 hours, were assessed for inflammation and organ damage; positron emission tomography was used to assess [^124^I]I-CBG biodistribution.

CLP mice developed septic shock leading to multi-organ failure and 58% mortality. CBG therapy reduced mortality to 17% (a relative decrease of 72%), reduced hypotension duration by 75%, and lowered organ damage markers. CBG transiently suppressed the pro-inflammatory cytokine peak at 12 hours (45%-59%) and markedly augmented anti-inflammatory interleukin-10 and interferon-β1 (2-fold to 96 hours). The decrease in corticosterone alongside this profile suggests an intrinsic anti-inflammatory response. Combined with PET-confirmed [^124^I]I-CBG targeting to the injury site, these data suggest CBG survival benefits are due to targeted delivery or direct immunomodulation.

While host responses involve a complex interplay of neuroendocrine and metabolic factors, our findings demonstrate marked improvements in disease progression and mortality with CBG therapy in murine-modeled septic shock. These results provide a strong impetus for a study of CBG therapy in patients with septic shock.

Sepsis is a life-threatening condition arising when the body's uncontrolled immune response to bacterial, fungal, parasitic, or viral infection causes widespread inflammation and organ damage (1). In 2020, sepsis was responsible for 20% of all global deaths (2), or more than 20 deaths per minute (3). This condition places a substantial economic burden on healthcare systems, with an estimated annual cost of AUD 842 million in Australia (4), USD 62 billion in the USA (5), and an average cost of USD 38 298 per patient (6).

Sepsis can escalate to septic shock, a condition the World Health Organization recognizes as a global health priority due to its severe cellular, metabolic, and circulatory dysfunction (1). This progression significantly increases mortality rates, reaching 33% to 42% in developed nations (7). The incidence of septic shock is expected to rise with advances in invasive surgery, implantable devices, and immunosuppressive therapies. Standard treatment for septic shock includes antimicrobials, fluid resuscitation, and vasopressors (8). In cases of refractory shock, glucocorticoids are added; however, glucocorticoid therapy frequently fails, offering limited 28-day survival benefits (9). Despite the high mortality and treatment failure rates, there have been no new septic shock therapies implemented into clinical practice for decades, underscoring a critical unmet medical need.

The principal glucocorticoid hormones, cortisol in humans and corticosterone in mice, circulate largely bound to proteins: approximately 80% to corticosteroid-binding globulin (CBG, encoded by the hepatic *SERPINA6* gene), 15% to albumin, and 5% as unbound, free hormone (10). In healthy individuals, plasma CBG concentrations typically range from 450 to 600 nmol/L (11).

Targeted glucocorticoid delivery is achieved through 2 distinct mechanisms. First, neutrophil elastase cleaves the reactive center loop of CBG, causing an irreversible conversion to a low-affinity form; this liberates cortisol, increasing interstitial fluid concentrations by 4-fold (12, 13). This cleavage event is structurally regulated by 6 glycosylation sites in the 383-amino acid protein; specifically, glycan occupancy at Asn347 acts as a variable steric inhibitor of this elastase activity at the Val344-Thr345 cleavage site (14). Second, pyrexia and acidosis induce a reversible release of cortisol while preserving CBG function, complementing the cleavage mechanism to target delivery to injured tissues in an inflammation-dependent manner (13). Murine CBG shares 60% sequence identity with human CBG, preserving the serpin tertiary structure. Despite sequence variations at the neutrophil elastase cleavage site (Val344-Thr345 in humans, Pro344-Pro345 in mice) and near Asn347, mouse CBG is predicted to maintain critical functions in glucocorticoid transport and the physiological stress response (15).

During septic shock, high concentrations of inflammatory cytokines, tumor necrosis factor α (TNFα), interleukin (IL)-1 (16), and IL-6 (17) inhibit hepatic CBG synthesis. Reduced circulating CBG leads to elevated free cortisol concentrations, which are strongly linked to increased mortality (18, 19). Our research has demonstrated that patients with septic shock and CBG below the lowest tertile on day 1 of septic shock admission (200 nmol/L) face a significantly increased intensive care unit (ICU) mortality (33% vs 14%, odds ratio 3.2) with CBG depletion the sole, potentially directly reversible, independent risk factor for septic shock mortality. Furthermore, patients with plasma CBG concentrations above 200 nmol/L required less ventilatory and vasopressor support in the ICU compared to those with CBG below 200 nmol/L (10, 20).

Together, these findings highlight CBG as a potential innovative and evidence-based therapy to safeguard or restore damage-based delivery of anti-inflammatory cortisol. Using a murine model of high-grade cecal ligation and puncture (CLP)-induced sepsis with wireless carotid telemetry, this study aimed to investigate the utility of CBG therapy, to provide the first fundamental knowledge on CBG pharmacokinetics, pharmacodynamics, and molecular targeting, and to evaluate the capacity of CBG therapy to reduce sepsis disease burden and mortality.

## Methods

### Ethics

This study was approved by the SAHMRI Animal Ethics Committee (as SAM-21-024) and carried out in compliance with the Australian Code for the Care and Use of Animals for Scientific Purposes (8th Edition) and the ARRIVE guidelines (21).

### Animals

Adult (10-12 weeks old) male C57BL/6 mice (n = 182) bred in SAHMRI Bioresources were housed individually in a 12:12 hour light-dark cycle (06:00 lights on, 18:00 lights off) under constant temperature (22 ± 0.5 °C) and humidity (40%-60%) with ad libitum access to water and a standard chow diet (13 kJ/g: 24% from protein, 18% from fat, 58% from carbohydrates; #2918, Teklad Global Diet, USA). To minimize circadian variability, all surgeries were completed during the early-light phase (08:30-10:30).

### Surgical procedures

#### Carotid telemetry

Mice were fasted for 2 hours (06:30-08:30), then anesthetized with 2.5% isoflurane in 0.5 L/min oxygen. A 1-cm incision was made 1 to 2 mm lateral to the trachea, the left common carotid artery was located, isolated with a 6-0 silk ligature (#18020-60; Fine Science Tools, Foster City, CA, USA) at the cephalic bifurcation, and a temporary microvascular clamp (#RS-5440; Roboz, USA) was placed below. A transverse incision (microdissection scissors #RS-5618; Roboz, USA) was made into the artery below the tie, and the catheter of an HD-X10 telemetry (#270-0171-002X; Data Science International, USA) was advanced 1 to 2 mm into the aortic arch (position standardized in age- and weight-matched mice) upon clamp removal. The catheter was secured with 2 silk ligatures, the telemetry body advanced subcutaneously along the right flank, and the incision closed using interrupted nonabsorbable polypropylene sutures (#PM196016F11M; eSutures, Mokena, IL, USA). Mice received 1 mL warmed 0.9% (w/v) sterile saline and 0.1 mg/kg buprenorphine (#TemVet, Troy Animal Healthcare, Glendenning, NSW, Australia) via subcutaneous injection, then recovered for 4 days in individually ventilated cages (IVCs) with wetted food pellets, enrichment, and 24-hour under-cage warming; daily monitoring included weight and blood pressure measurements.

#### High-grade cecal ligation and puncture

Mice were fasted for 2 hours (06:30-08:30), then anesthetized with 2.5% isoflurane in 0.5 L/min oxygen. A midline laparotomy was performed, the cecum located, exteriorized, then ligated with 4-0 silk (#A303H, Ethicon, Australia) 17 to 18 mm from the cecal tip. The cecum was punctured through-and-through twice with a 21G needle, a 1-mm column of feces extruded to assure patency, then the cecum was returned to position and the laparotomy closed with sutures (eSutures) and 7-mm wound clips (RS-9258, Roboz, Gaithersburg, MD, USA). Mice received 1 mL of warmed 0.9% (w/v) sterile saline, 0.1 mg/kg buprenorphine (Troylab), and 1 mg/100 g enrofloxacin (#1008343, Elanco GmbH, Cuxhaven, Germany) via subcutaneous injection, then returned to individual home IVCs with wetted food pellets, enrichment, and 12-hour under-cage warming.

### Postoperative care regimen

Mice were monitored and scored for body condition, real-time severe hypothermia (below 30 °C) or hyperthermia (above 38 °C), weight loss, reduced mobility, diarrhea, abdominal distension, labored breathing, or loss of righting reflex for a validated cumulative disease index (CDI) score (22) every 8 hours (07:00, 15:00, 23:00) for 4 days; the presence of labored breathing or loss of righting reflex was the threshold for early euthanasia. Mice received 0.1 mg/kg buprenorphine thrice daily by subcutaneous injection (07:00, 15:00, 23:00) for 2 days and 1 mL of warmed 0.9% (w/v) sterile saline twice daily (07:00, 23:00) for 4 days; enrofloxacin was not administered in the postoperative period.

### Survival studies

Initial dose-finding studies were performed using mouse recombinant CBG protein (His Tag, Sino Biological, Beijing, China; #50314-M08H) to establish a regimen that normalized plasma CBG concentrations in CLP mice to those in healthy, unoperated controls (n = 28, see Supplementary Methods (23)). To assess the therapeutic effects of CBG on sepsis progression, morbidity, and mortality, a refined dose strategy was implemented in CLP mice equipped with telemetry. An optimal CBG dose of 3.5 mg/kg was administered via intravenous (tail vein) injection at the 6-hour median onset of physiological decline, marked by hypotension and initial CBG decline, followed by a second dose of 2.5 mg/kg at 30 hours (n = 12); control CLP mice received volume-matched vehicle (200mM mannitol and 100mM trehalose in 0.9% (w/v) sterile saline) via tail vein injection (150 µL at 6 hours, 100 µL at 30 hours, n = 24). All mice received postoperative care as above, with the addition of tail vein blood sampling (20 µL) for CBG and lactate concentrations at 48 hours (clock time 09:00) prior to CLP, and at 12 (21:00), 30 (15:00), 54 (15:00), and 78 hours (15:00) post-CLP.

Surviving mice at day 4 (09:00) were fasted for 2 hours then anesthetized with 5% isoflurane in oxygen, with terminal cardiac bloods collected into EDTA tubes (1 mL, #450531, Interpath, Somerton VIC Australia) centrifuged at 12 000*g* for 15 minutes, and stored at −80 °C; bloods were collected from unfasted mice that met early euthanasia criteria at any point prior to 96 hour.

### Time course studies

A longitudinal assessment of CBG effects on sepsis progression and plasma biomarker profiles was undertaken in CLP mice equipped with telemetry and dosed with optimal CBG as above. CLP mice were randomized to administration of intravenous (tail vein) CBG (n = 40) or vehicle (n = 40), then to humane killing at 12, 24, 48, and 96 hours post-CLP (n = 10 per timepoint) with 2-hour fasting and blood collection as above; a subset of mice were humanely killed at 6 hours post-CLP to serve as pre-intervention controls. We measured plasma biomarkers in a cohort of age-matched unoperated male C57BL/6 mice (n = 6 per timepoint) to establish a reference daily range and mean, with sample collection at 0, 6, 12, and 24 hours, corresponding to clock times of 09:00, 15:00, 21:00, and 09:00. As sham-operated mice exhibit only transient increases in these biomarkers (which remain within the daily range (24, 25)), they were not used as controls in this study.

### Plasma analyses

Plasma concentrations of total corticosterone (#80556, Crystal Chem, Elk Grove Village, IL USA, RRID: AB 3677403), albumin (#80630, Crystal Chem, RRID: AB-3677402), and tissue hypoxia marker lactate, (#AB65331, Abcam plc, Cambridge, UK), and organ damage markers of cardiac troponin-I (#LS-F4165, LS Bio Newark, CA USA, RRID: AB 3677397), renal cystatin-C, (#AB119590, Abcam, RRID: AB 3677396), liver alanine transaminase (ALT; #AB282882, Abcam, RRID: AB 3094649), and aspartate aminotransferase (AST; #AB263882, Abcam, RRID: AB 3094650) were determined using commercial enzyme-linked immunosorbent assay (ELISA) kits. Free corticosterone was calculated using Coolen's equation (26). Plasma cytokines were determined via a customized MILLIPLEX® Mouse High Sensitivity T Cell Magnetic Bead Panel for TNFα, IL-6, IL-10, IL-12, macrophage inflammatory protein 2 (MIP-2), and interferon-β1 (IFN-β1) (#MHSTCMAG-70 K, Merck KGaA, Darmstadt, Germany, RRID: AB 3677398). All assays were conducted as per the manufacturer's instructions.

Total plasma CBG was quantified using a custom in-house sandwich ELISA. Briefly, 96-well plates (#82.1581.210, Sarstedt, Numbrecht, Germany) were coated with 100 μL of rabbit polyclonal anti-CBG antibody (#50314, Sino Biological, Beijing, China) diluted 1:1000 in coating buffer (1.9 g Na_2_CO_3_, 2.9 g NaHCO_3_ in 1 L of distilled water, pH adjusted to 9.6) and incubated for 18 hours at 4 °C with shaking (200 rpm). After washing (3 × 200 μL) in phosphate-buffered saline ([PBS] ThermoFisher Scientific, Waltham, MA, USA) with 0.05% Tween 20 (PBS-T), wells were blocked with PBS-T containing 1% gelatin for 30 minutes, followed by another wash. Mouse plasma samples (100 μL), diluted 1:1000 in PBS-T with 0.1% gelatin, were then added and incubated for 2 hours at room temperature. The plate was washed (6×) and 100 μL of biotinylated rabbit polyclonal anti-CBG antibody (1:500 in PBS-T with 0.1% gelatin; Sino Biological) was added per well for 1 hour at room temperature. After washes (6×), 95 μL of streptavidin alkaline phosphatase (#016-050-084, Jackson ImmunoResearch, West Grove, PA, USA) diluted 1:1000 in PBS-T with 0.1% gelatin was added and incubated for 30 minutes at room temperature. After a final wash, 100 μL of QUANTI-Blue™ (#rep-qbs, InvivoGen, San Diego, CA, USA) was added per well for 1 hour with intermittent shaking. Absorbance was then measured at 640 nm using a BioTek Synergy HTX Multimode reader (Agilent Technologies, Santa Clara, CA, USA).

### Radiolabeled CBG synthesis

Two Pierce^TM^ Iodination beads (ThermoFisher Scientific) were washed with PBS (1 mL), then dried on filter paper. 90 MBq of [^124^I]I-NaI in 20 mM NaOH solution (Austin Health, Heidelberg, VIC, Australia) was diluted to 200 µL with PBS in a 1.5-mL Protein LoBind® Eppendorf tube (Eppendorf AG, Hamburg, Germany). All radioactivity measurements were performed using a Capintec CRC-55tR Dose Calibrator (Mirion Medical, Florham Park, NJ, USA) with calibration setting #700. The iodination beads were added, and the reaction was shaken at room temperature (350 rpm) for 5 minutes. CBG (100 µg, Sino Biological) was dissolved in PBS (200 µL), added to the reaction vessel, and shaken at room temperature (350 rpm) for 30 minutes. The iodination beads were washed with PBS (2 × 50 µL) to recover bound protein. The labeled protein was purified via an Amicon Ultra (0.5 mL, 10 K) spin-filter (Merck KGaA, Darmstadt, Germany) per manufacturer's instructions and washed with PBS (3 × 400 µL) to remove unbound [^124^I]I-NaI. The protein was recovered as directed and transferred to a 2-mL microcentrifuge tube (Corning Inc., Corning, NY, USA). Remaining protein on the spin-filter was rinsed with PBS (150 µL) and combined with the purified protein; 27.9 MBq of [^124^I]I-CBG was obtained and formulated with 0.9% (w/v) sterile saline (600 µL) for a biodistribution cohort in control C57BL/6 mice (total volume approximately 750 µL). Instant thin-layer chromatography (iTLC) analysis was performed using a 2-μL sample aliquot on an Agilent iTLC-SG plate (Agilent Technologies) with 20mM citric acid as the mobile phase. iTLC plates were analyzed on a LabLogic ScanRam (LabLogic Systems, Chantilly, VA, USA) with PMT NaI 1-inch detector and processed on LabLogic Systems Laura (ver. 4.2.11.129), resulting in a radiochemical purity of 96.7% [^124^I]I-CBG (Rf = 0.0-0.1) with 3.3% [^124^I]I-NaI (Rf = 0.7). Molar activity of 14 MBq/µmol was determined via high-performance liquid chromatography (HPLC) (Shimadzu LC-20AD with SPD-M30A photo-diode array [Shimadzu Corporation, Kyoto, Japan] and radio-detector [LabLogic Flowram with PMT NaI 1-inch]). Samples (20 μL) were manually injected and analyzed on a 3-μm Biozen dSEC-2 column (300 × 7.8 mm, Phenomenex Inc., CA, USA) with isocratic 200mM ammonium acetate at a flow rate of 1 mL/min. [^124^I]I-CBG Rt = 8.2 minutes and [^124^I]I-NaI Rt = 11.2 minutes.

Radiosynthesis was scaled (2x) for the CLP-sepsis cohort to yield 53.2 MBq of [^124^I]I-CBG, which was formulated with unlabeled CBG (800 µg) in 0.9% (w/v) sterile saline (1.2 mL; total volume 1.5 mL). The iTLC analysis was performed as above, resulting in radiochemical purity of 91.0% [^124^I]I-CBG (Rf = 0.0-0.1) with 9.0% [^124^I]I-NaI (Rf = 0.7). The iTLC analysis at 48 hours post-synthesis resulted in a radiochemical purity of 90.0% [^124^I]I-CBG (Rf = 0.0-0.1) with 10.0% [^124^I]I-NaI (Rf = 0.7). Molar activity of 3 MBq/µmol was determined via HPLC as described above.

### Positron emission tomography

#### Control mice

Adult (10-12 weeks old) male C57BL/6 mice (unoperated, n = 6) were anesthetized with 2% isoflurane in 0.5 L/min air, then 150 µL of [^124^I]I-CBG (2.4 ± 0.1 MBq; 10.7 ± 0.5 µg dose/animal), followed by 50 µL of 0.9% (w/v) sterile saline, was administered via intravenous (tail vein) injection, before their return to individual IVCs. Half of the mice were allocated to imaging in a prone, single bed position (40-mm offset, Table 1) in a Bruker Albira positron emission tomography/single photon emission computed tomography (PET-SPECT) small animal scanner (Bruker BioSpin GmbH, Ettlingen, Germany) immediately (0 minutes; n = 3) or 90 minutes post-injection (n = 3). Mice were then re-anesthetized and re-imaged at 24, 48, and 72 hours after injection.

**Table 1:** Scanning protocol in unoperated mice

Time post injection	Frame duration	Type; purpose
**0 minutes** (SUV_0-30 minutes_)		
0-2 minutes	10 second × 12 frames	Dynamic; early blood distribution
2-10 minutes	30 second × 16 frames	Dynamic; tissue perfusion phase
10-30 minutes	1 minute × 20 frames	Dynamic; metabolism and uptake phase
**90 minutes** (SUV_90-120 minutes_)		
90-120 minutes	5 minutes × 6 frames	Dynamic; late-phase kinetics
**6 hours** (SUV_6 hr_)	15 minutes × 1 frame	Static; biodistribution and clearance
**24 hours** (SUV_24 hr_)	15 minutes × 1 frame	Static; biodistribution and clearance
**48 hours** (SUV_48 hr_)	15 minutes × 1 frame	Static; biodistribution and clearance
**72 hours** (SUV_72 hr_)	15 minutes × 1 frame	Static; biodistribution and clearance

Abbreviation: SUV, standardized uptake value.

#### CLP-sepsis mice

Adult male C57BL/6 mice (10-12 weeks old, n = 10) underwent high-grade CLP as above, then at 7.5 hours (n = 5) or 12 hours (n = 5) post-CLP, a 140 µL solution of radiolabeled [¹²⁴I]I-CBG (2.1 ± 0.2 MBq; 4.2 ± 0.4 mg/mouse) and unlabeled CBG (41.5 ± 3.9 mg/mouse), followed by 60 µL of 0.9% (w/v) sterile saline, was administered via an intravenous tail vein injection; due to their health status, CLP mice were not anesthetized for this injection. Mice were anesthetized and PET imaged as above at 7.5-hour or 12-hour time points (Table 2), with survivors re-anesthetized and re-imaged to profile [¹²⁴I]I-CBG uptake at 24 hours (n = 10) and 48 hours (n = 7) post-CLP.

**Table 2:** Scanning protocol in CLP-CBG mice

Time post injection	Frame duration	Type; purpose
**7.5 hours** (SUV_90-105 minutes_)		
90-105 minutes	5 minutes × 3 frames	Dynamic; Late-phase kinetics
**12 hours** (SUV_6 hr_)	15 minutes × 1 frame	Static; Biodistribution and Clearance
**24 hours** (SUV_18 hr_)	15 minutes × 1 frame	Static; Biodistribution and Clearance
**48 hours** (SUV_42 hr_)	15 minutes × 1 frame	Static; Biodistribution and Clearance
**72 hours** (SUV_66 hr_)	15 minutes × 1 frame	Static; Biodistribution and Clearance

Abbreviation: SUV, standardized uptake value.

### Positron emission tomography image analysis

PET scans were reconstructed using the OSEM protocol from ParaVision 360 Software (Bruker BioSpin GmbH), normalized and corrected for attenuation, scattering, and decay, and analyzed as dynamic (3 frames × 5 minutes) or total (15 minutes) emission data. PET images were visualized and analyzed using PMOD (Version 4.2; PMOD Technologies LLC, Zurich, Switzerland). Tracer uptake (%ID/cc), SUV_avg_, SUV_max_ values in CLP mice were compared with volumes of interest (VOIs) from control C57BL/6 mice. Values were calculated using the volumes of interest analysis in PMOD Software (see Fig. S1 (23)).

### Statistical analyses

Survival in CLP mice was evaluated using a 4-day Kaplan-Meier curve. All data were assessed for normality via the Shapiro-Wilk test. Weight change, CDI score, hemodynamics, and plasma biomarkers were compared between surviving and early euthanasia mice using a multivariate analysis of variance in SPSS v29.0 (IBM Corporation, NY, USA), with time included as a between-mice effect. CDI score, weight changes, and plasma time course data were analyzed using a 2-way ANOVA with Tukey's post hoc analysis using GraphPad Prism v10.0 (Dotmatics, Boston, MA, USA).

PET imaging data were analyzed using PMOD v4.3 (PMOD Technologies, Zurich, Switzerland). Standardized uptake values (SUV) were extracted from defined organ VOIs across timepoints. All SUV data were assessed for normality using the Shapiro-Wilk test. Longitudinal tracer uptake was analyzed using a mixed-effects model with uncorrected Fisher's least significant difference test for multiple comparisons. Correlation analyses between organ tracer uptake and survival outcomes were performed using Pearson's *r* correlation.

## Results

### Survival series (tail bleeds; longitudinal)

**Figure 1:**
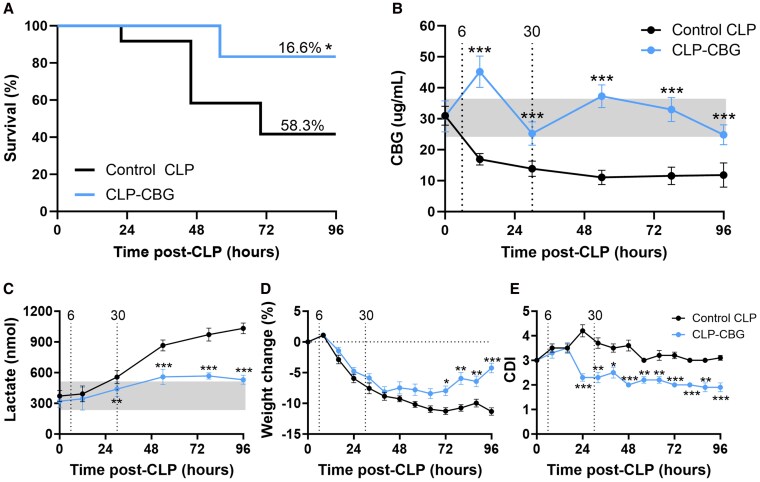
CBG therapy improves survival, morbidity, and safeguards plasma lactate in CLP-sepsis. Survival series data (0-96 hours) showing (A) Kaplan-Meier survival in control CLP (n = 24) and CLP-CBG mice (n = 12), (B) plasma CBG, (C) plasma lactate, (D) body weight, and (E) cumulative disease index (CDI, ie, unoperated mice score zero); (B-E) n = 10 control CLP, n = 10 CLP-CBG mice. Light gray boxes represent the daily range of plasma CBG, lactate in unoperated mice. Vertical dotted lines mark times of vehicle (control CLP, black) or CBG administration (CLP-CBG, blue). Data are mean ± SEM; *P ≤ .05, **P ≤ .01, ***P ≤ .001 (CLP-CBG vs control CLP).

The impact of CBG therapy on mortality, plasma CBG and lactate concentrations, weight, and CDI scores is detailed in Fig. 1; prestudy CBG dose-range findings are presented in Fig. S2 (23). Vehicle-treated high-grade CLP mice (control CLP) showed 58.3% mortality (14/24) at day 4, while CBG-treated high-grade CLP mice (CLP-CBG) showed a 71.5% reduction in mortality to 16.6% (2/12, absolute risk reduction 41.7%, *P* = .022 Fig. 1A). In the vehicle-treated group, 2, 8, and 4 mice required early euthanasia at 22, 46, and 70 hours, respectively, whereas only 2 mice in the CBG-treated group required this procedure (both at 56 hours).

Plasma CBG concentration in the survival cohort declined below their 48-hour pre-CLP mean from 12 hours (45%, *P* ≤ .001) to 96 hours (61%, *P* ≤ .001) with a nadir at 54 hours (64%) in control CLP mice. Intravenous CBG administration at 6 and 30 hours post-CLP maintained plasma CBG within the daily CBG range in unoperated mice in CLP-CBG mice, except for an increase at 12 hours (47%, *P* = .001; treatment effect *P* = .005; Fig. 1B). Across both cohorts, lower plasma CBG was consistently associated with early euthanasia (*P* < .001; Table 3). Plasma lactate increased steadily in control CLP mice from 54 hours (30%, *P* = .002) to 96 hours (69%, *P* ≤ .001) relative to their 48-hour pre-CLP mean, while CLP-CBG mice maintained plasma lactate concentrations within the daily lactate range in unoperated mice at all time points (Fig. 1C); irrespective of treatment, mice requiring early euthanasia exhibited higher plasma lactate (29%, *P* ≤ .001; Table 3).

**Table 3:** Plasma biomarkers for CLP-sepsis survivors and nonsurvivors

Biomarker	Survivors		*p^1^*	Nonsurvivors	*p^2^*
	Control CLP	CLP-CBG		Control CLP	
CBG (µg/mL)	11.8 ± 1.2	24.8 ± 1.1	**≤.001**	4.7 ± 0.4	**≤.001**
Lactate (nmol)	1032.6 ± 22.9	530.1 ± 13.9	**≤.001**	1324.1 ± 29.6	**.003**
IL-1β (ng/mL)	1.1 ± 0.1	0.6 ± 0.1	**.028**	3.75 ± 0.2	**≤.001**
IL-6 (ng/mL)	9.5 ± 0.7	4.4 ± 0.7	**.001**	18.2 ± 1.1	**≤.001**
IL-12 (ng/mL)	1.8 ± 0.2	1.1 ± 0.1	**.018**	4.4 ± 0.7	**≤.001**
MIP-2 (ng/mL)	1.0 ± 0.2	0.7 ± 0.1	.475	1.23 ± 0.26	.725
TNFα (ng/mL)	0.6 ± 0.05	0.5 ± 0.09	.962	1.63 ± 0.1	**≤.001**
IL-10 (ng/mL)	6.3 ± 0.8	13.0 ± 0.9	**≤.001**	12.4 ± 0.5	**≤.001**
IFN-β1 (ng/mL)	0.08 ± 0.01	0.58 ± 0.04	**≤.001**	1.61 ± 0.08	**≤.001**
T-Cort (ng/mL)	310.6 ± 34.6	197.1 ± 21.4	.156	524.3 ± 22.4	**≤.001**
F-Cort (ng/mL)	50.24 ± 6.4	26.6 ± 3.4	**.045**	125.1 ± 6.2	**≤.001**
Albumin (mg/mL)	9.3 ± 0.7	19.2 ± 1.7	**≤.001**	4.2 ± 0.3	**≤.001**
Cystatin-C (ng/mL)	555.4 ± 15.8	312.0 ± 27.7	**≤.001**	579.1 ± 12.6	.192
Troponin-I (ng/mL)	74.3 ± 4.2	33.2 ± 1.8	**≤.001**	89.2 ± 3.1	**.044**
ALT (ng/mL)	528.7 ± 36.1	648.7 ± 57.2	.458	950.4 ± 44.3	.216
AST (ng/mL)	2062.8 ± 236.1	1385.8 ± 117.4	.947	5479 ± 1	**.046**

Data (mean ± SEM) were time adjusted for death; *p^1^* = 96 hour adjusted CLP-CBG survivors vs control CBG survivors (each n = 10), *p^2^* = 96 hour adjusted control CBG survivors (n = 10) vs nonsurvivors (n = 14); bold *P* values are significantly different.

Abbreviations: ALT, alanine aminotransaminase; AST, aspartate aminotransferase; CBG, corticosteroid-binding globulin; F-Cort, free corticosterone; IFN-β1, interferon-β1; IL-10, interleukin 10; IL-12, interleukin-12; IL-1β, interleukin-1β; IL-6, interleukin-6; MIP-2, macrophage inflammatory protein-2; T-Cort, total corticosterone; TNFα, tumor necrosis factor α.

Body weight decreased in control CLP mice from 32 hours (7.5 ± 0.9%, *P* = .008) to 96 hours (11.3 ± 0.6%, *P* ≤ .001) relative to pre-operative levels, while CLP-CBG mice had decreased weight from 40 hours (8.1 ± 0.8%, *P* = .002) to 72 hours post-CLP (7.9 ± 0.8%, *P* = .003) before recovery at 96 hours (treatment effect, *P* ≤ .001; Fig. 1D). Weight loss was similar in surviving mice and mice requiring early euthanasia in their respective groups. CDI score peaked at 24 hours (∼4) in control CLP mice before declining to stabilize through to 96 hours (∼3). The CDI score in CLP-CBG mice showed an earlier (16 hours), lower peak (∼3.5), and then declined further (to ∼2) from 24 to 96 hours (24-hour *P* = .003; 96-hour *P* = .024), indicating a CBG effect to lower CDI (treatment effect *P* ≤ .001; Fig. 1E). CDI was higher in all CLP nonsurvivors compared to survivors (2.6-fold, *P* ≤ .001).

#### Mean arterial pressure

**Figure 2:**
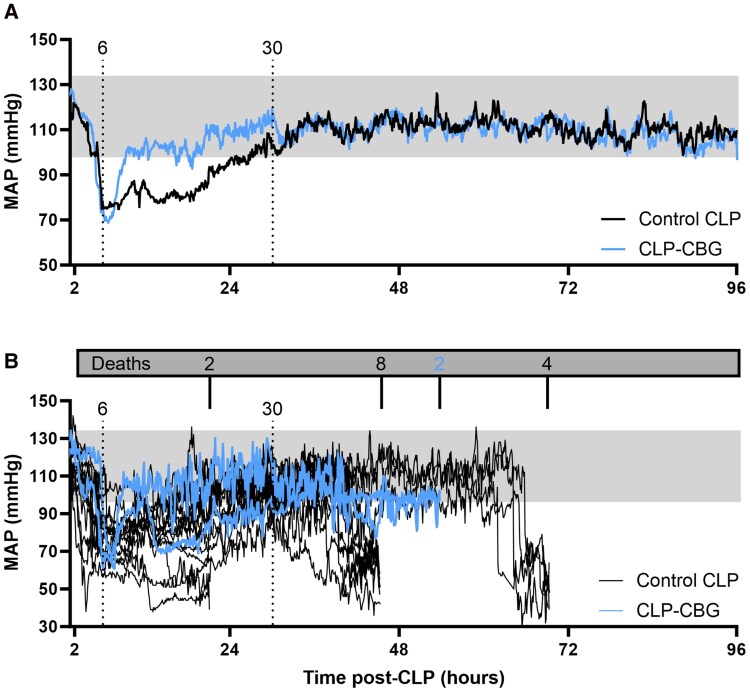
CBG therapy improves hemodynamic recovery in CLP-sepsis. Survival series mean arterial blood pressure (MAP) average in (A) control CLP and CLP-CBG survivors to 96 hours, and (B) individual MAP records in CLP nonsurvivors (in-cage death, n = 2 or early euthanasia, n = 14 prior to 96 hours); n = 10 control CLP, n = 10 CLP-CBG. Vertical dotted lines mark times of vehicle (control CLP, black) or CBG administration (CLP-CBG, blue); Dark gray box indicates number of deaths and times; light gray region represents the MAP range in the 48 hours preceding CLP.

Real-time mean arterial pressure (MAP) is illustrated in Fig. 2. Isolated episodes of hypotension, defined as a 40-mmHg decrease in MAP within 10 minutes from the 48-hour pre-CLP mean (27), featured in all CLP mice in the period 3.8 to 7.1 hours, with onset at 4.9 ± 0.4 hours (prior to any intervention). In control CLP survivors, the first hypotensive episode lasted for 7.5 ± 1.5 hours, reaching a nadir of 61 ± 4 mmHg (Fig. 2A), but was longer in control CLP nonsurvivors, lasting 11.6 ± 1.1 hours with a nadir of 59 ± 6 mmHg (*P* = .042, Fig. 2B). In contrast, CLP-CBG survivors experienced a shorter first episode lasting 3.4 ± 0.3 hours, less than that in control CLP survivors (*P* = .005) and nonsurvivors (*P* ≤ .001), despite a similar MAP nadir (62 ± 3 mmHg, Fig. 2A); statistical comparisons for CLP-CBG nonsurvivors could not be completed due to the small number of mice (2/12). Of the CLP control mice that experienced episodes of hypotension, half (12/24) had 2 or more episodes, which were lethal. The other half (12/24) had a single episode, and most survived (10/12). In contrast, only 3 CLP-CBG mice (3/12) exhibited multiple episodes of hypotension, which were lethal in 2 mice; the remaining CLP-CBG mice (9/12) had a single episode, and all survived.

Septic shock, defined as hypotension concurrent with an elevation in plasma lactate 20% above the daily range mean of lactate in unoperated mice, developed in 58% (14/24) of control CLP mice, proving uniformly lethal. The onset of septic shock varied, at 13.5 ± 0.9 hours (2 mice), 39.3 ± 1.3 hours (8 mice), and 64.5 ± 0.8 hours (4 mice) and lasted 6.7 ± 0.8 hour until early euthanasia, with a final MAP of 53 ± 3 mmHg (Fig. 2B). In contrast, none of the CLP-CBG mice developed septic shock, and although 2/12 underwent early euthanasia at 56 hours coincident with elevated lactate, their final MAP remained above the threshold of hypotension (93, 96 mmHg, Fig. 2B).

#### Heart rate

**Figure 3:**
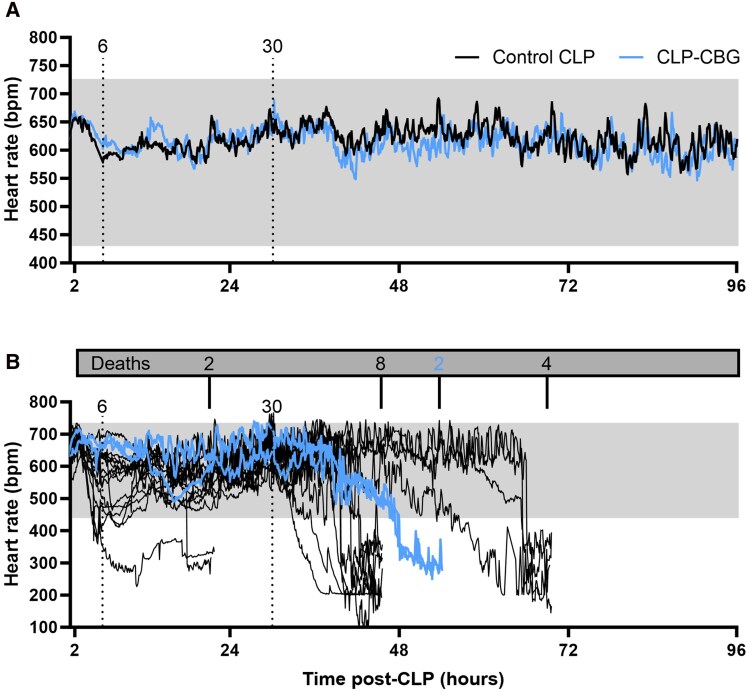
CBG therapy does not alter heart rate responses in CLP-sepsis. Survival series heart rate (bpm) average in (A) control CLP and CLP-CBG survivors to 96 hours, and (B) individual MAP records in CLP nonsurvivors (in-cage death, n = 2 or early euthanasia, n = 14 prior to 96 hour); n = 10 control CLP, n = 10 CLP-CBG. Vertical dotted lines mark times of vehicle (control CLP, black) or CBG administration (CLP-CBG, blue); Dark gray box indicates number of deaths and times; light gray region represents the MAP range in the 48 hours preceding CLP.

Real-time heart rate is illustrated in Fig. 3. A 100-bpm heart rate decline within 10 minutes from the 48-hour pre-CLP mean was not seen in any control CLP or CLP-CBG survivor, despite hypotension episodes (Fig. 3A). However, CLP-CBG mice showed a trend toward a faster heart rate compared to control CLP mice (*P* = .053), with a significant treatment × time effect (*P* = .006). In contrast, heart rate declined by 48% to 282 ± 11 bpm (*P* = .015) in most control CLP nonsurvivors (13/14), beginning 2.1 ± 0.5 hours after the MAP decline of septic shock, with a final heart rate of 215 ± 16 bpm; except for a single mouse, where the heart rate dropped to 251 bpm, beginning 4.3 hours prior to the MAP drop of septic shock, with a final heart rate of 201 bpm. Even without septic shock, both CLP-CBG nonsurvivors also exhibited a heart rate decline: a 52% decrease to 301 bpm at 48 hours, and a 50% decrease to 311 bpm at 49.5 hours post-CLP (Fig. 3B).

#### Temperature

**Figure 4:**
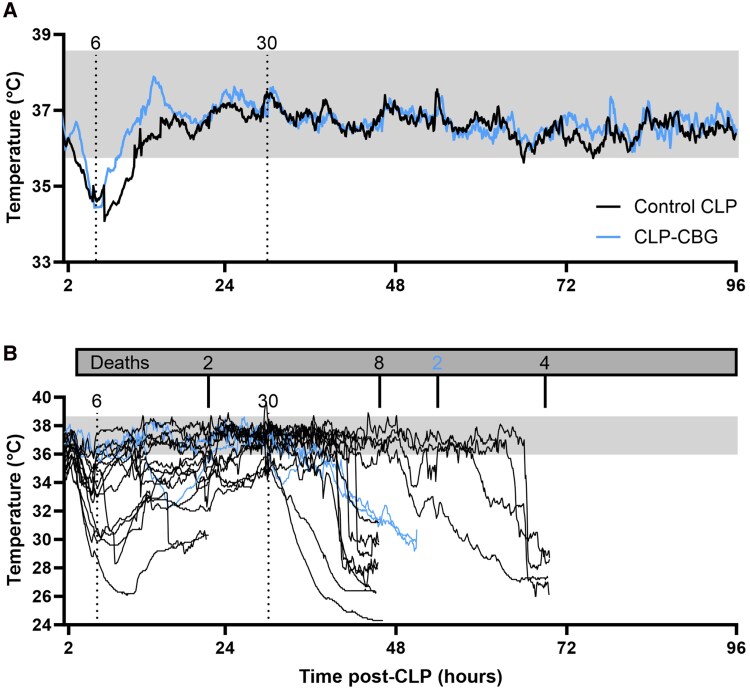
CBG therapy improves thermoregulation in CLP-sepsis. Survival series core temperature (°C) average in (A) control CLP and CLP-CBG survivors to 96 hour, and (B) individual temperature records in CLP nonsurvivors (in-cage death, n = 2 or early euthanasia, n = 14 prior to 96 hours); n = 10 control CLP, n = 10 CLP-CBG. Vertical dotted lines mark times of vehicle (control CLP, black) or CBG administration (CLP-CBG, blue); Dark gray box indicates number of deaths and times; light gray region represents the core temperature range in the 48 hours preceding CLP.

Real-time calibrated core temperature profiles are illustrated in Fig. 4. Mild hypothermia between 35.5 and 32 °C was observed in all CLP mice at 4.2 ± 0.3 hours post-CLP (prior to any intervention). This lasted 6.8 ± 0.9 hours in control CLP survivors and 10.3 ± 2.4 hour in nonsurvivors, with a nadir of 33.7 ± 0.4 °C and 32.6 ± 0.5 °C, respectively. In contrast, mild hypothermia in CLP-CBG survivors lasted 3.9 ± 0.6 hours, with a nadir of 34.3 ± 0.3 °C, and was shorter than that in control CLP survivors (*P* = .015) and nonsurvivors (*P* ≤ .001), indicating a significant CBG effect (*P* = .046). For both CLP-CBG nonsurvivors, mild hypothermia lasted 4.5 and 6.3 hours, with nadirs of 35.1 and 32.3 °C, respectively.

An onset of hypothermia (below 32 °C) was observed in all control CLP nonsurvivors at 3.9 hours (1/14), 15.9 hours (1/14), 33.5 hours (2/14), 41.0 ± 0.5 hours (6/14), 54.6 hours (1/14), and 66.1 hours (3/12), with most (10/14) beginning 1.9 ± 0.6 hours prior to the septic shock–related MAP decline, then progressing to severe hypothermia, below 30 °C, with a final temperature of 28.4 ± 0.5 °C at early euthanasia (Fig. 4B). The remaining control CLP nonsurvivors (4/14) exhibited an earlier temperature decline starting 9.9 ± 1.1 hours before the septic shock–related MAP decline, then progressed to severe hypothermia with a final temperature of 27.9 ± 1.3 °C at early euthanasia. Even without septic shock, both CLP-CBG nonsurvivors exhibited a similar temperature decline at 38.9 and 37.5 hours post-CLP, resulting in final temperatures of 30.7 and 29.8 °C at early euthanasia (Fig. 4B).

#### Activity

**Figure 5:**
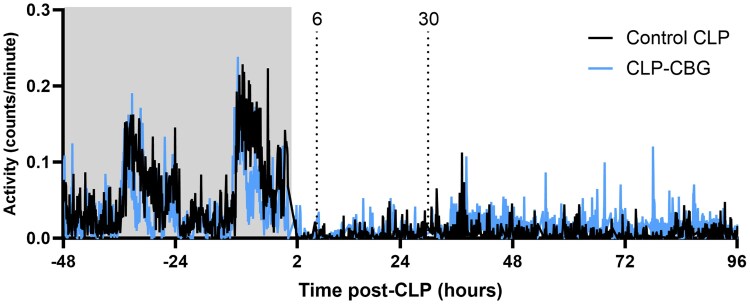
CBG therapy tends to increase activity in CLP-sepsis. Survival series activity (counts/min) average in control CLP and CLP-CBG survivors from 48 hours pre-CLP to 96 hours post-CLP; n = 10 control CLP, n = 10 CLP-CBG. Vertical dotted lines mark times of vehicle (control CLP, black) or CBG administration (CLP-CBG, blue); Light gray region highlights averaged activity in the 48 hours preceding CLP.

Real-time, in-cage activity is illustrated in Fig. 5. All mice showed decreased activity after CLP compared to the 48-hour pre-CLP period (87%, *P* ≤ .001), while CLP-CBG mice tended to increase activity from 40 hours post-CLP compared to control CLP mice (28%, *P* = .057, Fig. 5). Furthermore, all CLP nonsurvivors (control or CBG-treated) showed consistently decreased activity until their death, compared to CLP survivors (*P* = .029).

### Positron emission tomography imaging

**Figure 6:**
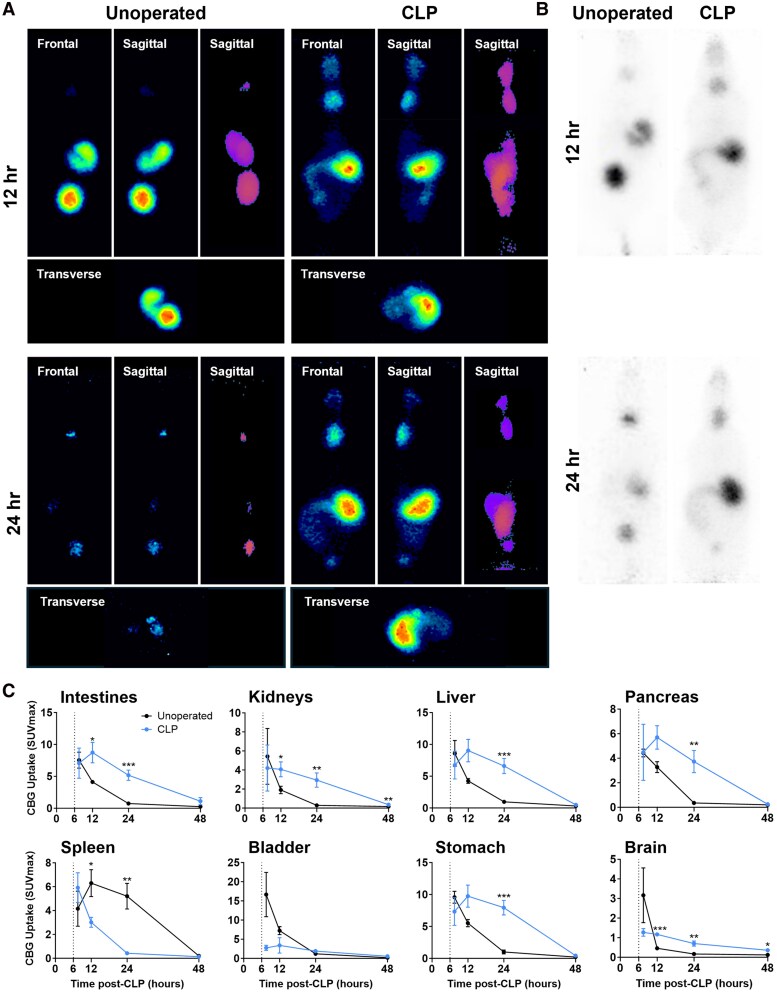
CBG localizes to multiple tissues in CLP-CBG mice. (A) Representative maximum intensity projection images (qualitative; second sagittal panel = solid projection showing labeled peri-gastric intestine (orange) adjacent to the stomach (purple)) and (B) maximum signal intensity (quantitative) images from unoperated and CLP mice. (C) Maximum standardized uptake value (SUVmax) of intravenous [^124^I]I-CBG (6-hour vertical dashed line) in unoperated (black lines) and CLP-CBG mice (blue lines) from 7.5 to 48 hours after CLP; n = 6 unoperated controls, n = 10 CLP-CBG. *P ≤ .05, **P ≤ .01, ***P ≤ .001 vs unoperated controls.

The biodistribution profile of [¹²⁴I]I-CBG is illustrated in Fig. 6 (see Fig. S2 for volume of interest atlas (23)). Longitudinal PET imaging revealed differential tracer uptake in unoperated and CLP-CBG mice from 12 to 48 hours. CBG uptake was similar in all VOIs in unoperated and CLP-CBG mice at 7.5 hours (*P* ≥ .05, Fig. 6A-6C). At 12 hours, tracer localization was prominent at the peri-ligature intestine in CLP-CBG mice and increased in the intestine (0.4008 mean diff, *P* = .042), kidneys (0.4739 mean diff, *P* = .002), pancreas (0.8937 mean diff, *P* = .029), and brain (0.1609 mean diff, *P* = .005) relative to unoperated mice (Fig. 6B). CBG uptake differences peaked at 24 hours, with persistent tracer accumulation at the peri-ligature intestine in CLP-CBG mice and increased uptake in the intestine (0.5260 mean diff, *P* = .007), kidneys (0.4008 mean diff, *P* = .042), liver (0.5448 mean diff, *P* = .002), pancreas (1.278 mean diff, *P* = .014), spleen (1.72 mean diff, *P* = .008), stomach (3.419 mean diff, *P* ≤ .001), and brain (0.1240 mean diff, *P* = .002, Fig. 6C). CBG uptake declined toward unoperated levels at 48 hours in CLP-CBG mice, although signal remained detectable in the intestinal region of individual mice (*P* ≥ .05).

Correlation analysis revealed that higher 24-hour pancreatic uptake was negatively associated with 48-hour survival (SUV_avg_  *r* = −0.656, *P* = .045; SUV_max_  *r* = −0.653, *P* = .041). In contrast, higher 12-hour kidney uptake was positively associated with survival (SUV_max_  *r* = 0.945, *P* = .015); no other organ showed significant correlation (all *P* ≥ .05).

### Time course series (cardiac blood)

#### CBG and corticosterone

**Figure 7:**
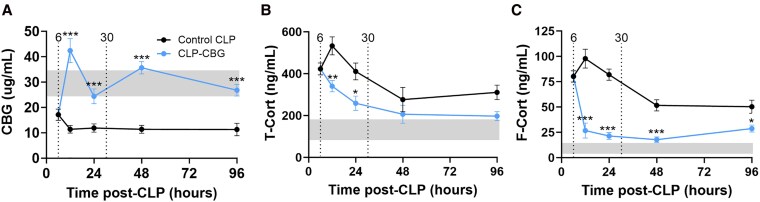
CBG therapy attenuates total and free plasma corticosterone in CLP-sepsis. Time course series plasma profiles of (A) CBG, (B) total (T-Cort), and (C) free corticosterone (F-Cort) in control CLP and CLP-CBG mice (each n = 10 per timepoint) from 6 to 96 hours post-CLP. Vertical dotted lines mark times of vehicle (control CLP, black) or CBG administration (CLP-CBG, blue); Light gray region highlights the daily range of CBG, total or free corticosterone concentrations in age-matched, unoperated mice (n = 6 each at 0, 6, 12, and 24 hours). Data are mean ± SEM; *P ≤ .05, **P ≤ .01, ***P ≤ .001 (CLP-CBG vs control CLP).

The profile of plasma CBG, total, and free corticosterone is illustrated in Fig. 7. Plasma CBG concentration declined in control CLP mice, prior to any intervention, from 6 hours (45%, *P* = .023) to 96 hours (61%, *P* ≤ .001) with a 48-hour nadir (63%, *P* ≤ .001) relative to the daily CBG range mean in unoperated mice. CBG therapy maintained plasma CBG concentrations within this range, except for an increase at 12 hours (37%, *P* = .024) with a significant CBG treatment (*P* ≤ .001) and treatment × time effect (*P* = .017; Fig. 7A).

Plasma total corticosterone increased from 6 hours in control CLP mice (2.2-fold, *P* = .002) prior to any intervention, peaked at 12 hours (2.8-fold, *P* ≤ .001), then declined but remained elevated to 96 hours relative to the daily range mean of total corticosterone in unoperated mice (1.6-fold, *P* = .032; Fig. 7B). In contrast, CLP-CBG mice exhibited a 56% decline in total corticosterone at 12 hours compared to control CLP mice (*P* ≤ .001), which then normalized within the daily range in unoperated mice beyond 24 hours, with a time effect (*P* ≤ .001) and CBG treatment effect trend (*P* = .059) (Fig. 7B). Plasma total corticosterone concentrations were higher in control CLP nonsurvivors compared to survivors (69%, *P* ≤ .001; Table 3).

Plasma free corticosterone increased at 6 hours in control CLP mice (6.3-fold, *P* ≤ .001), prior to any intervention, peaked at 12 hours (8.2-fold, *P* ≤ .001), then declined but remained elevated at, and beyond, 24 hours relative to the daily range mean of free corticosterone in unoperated mice (3.5-fold, *P* = .002). In contrast, free corticosterone was lower in CLP-CBG mice compared to control CLP mice beyond 12 hours (74%, *P* ≤ .001), then normalized within the daily range in unoperated mice with a significant CBG treatment (*P* ≤ .001), and time effect (*P* ≤ .001), and a time × treatment interaction (*P* ≤ .001; Fig. 7C). Free corticosterone concentrations were higher in all CLP nonsurvivors compared to survivors (149%, *P* ≤ .001; Table 3).

#### Cytokines

**Figure 8:**
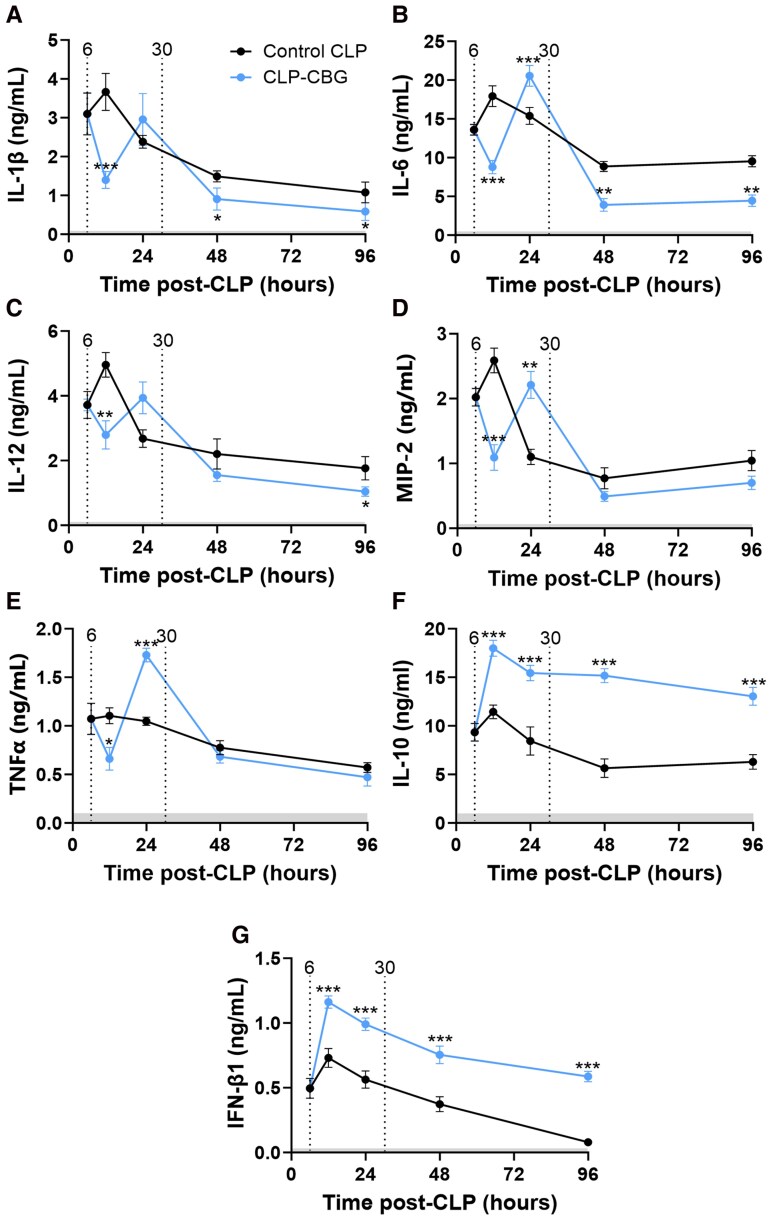
CBG therapy transiently attenuates pro-inflammatory cytokines and produces a sustained elevation in anti-inflammatory cytokines in CLP-sepsis. Time course series plasma profiles of (A) interleukin (IL)-1β, (B) IL-6, (C) IL-12, (D) macrophage inflammatory protein 2 (MIP-2), (E) tumor necrosis factor α (TNFα), (F) IL-10, and (G) interferon (IFN)-β1 in control CLP and CLP-CBG mice (each n = 10 per timepoint) from 6 to 96 hours post-CLP. Vertical dotted lines mark times of vehicle (control CLP, black) or CBG administration (CLP-CBG, blue); Light gray region highlights the daily range of IL-1β, IL-6, IL-12, MIP-2 or TNFα concentrations in age-matched, unoperated mice. Data are mean ± SEM; *P ≤ .05, **P ≤ .01, ***P ≤ .001 (CLP-CBG vs control CLP).

Plasma cytokine profiles, detailed in Fig. 8, were markedly elevated in control CLP mice. Prior to any intervention, IL-1β, IL-6, IL-12, MIP-2, TNFα, IL-10, and IFN-β1 increased at 6 hour by 61-fold, 84-fold, 37-fold, 21-fold, 9.7-fold, 45-fold, and 8.8-fold, respectively, relative to their daily range mean concentrations in unoperated mice (all *P* ≤ .001). These peaked at 12 hours, with an increase of 71-fold for IL-1β, 178-fold for IL-6, 164-fold for IL-12, 40-fold for MIP-2, 12-fold for TNFα, 70-fold for IL-10, and 17-fold for IFN-β1 (all *P* ≤ .001). Subsequently, IL-1β, IL-6, IL-12, MIP-2, TNFα, and IL-10 declined, but remained elevated to 96 hours (20-fold, *P* = .003; 94-fold, *P* ≤ .001; 57-fold, *P* = .002; 16-fold, *P* ≤ .001; 6.1-fold, *P* = .026, respectively; all time effects *P* ≤ .001). In contrast, IFN-β1 concentrations had normalized within the daily range of IFN-β1 in unoperated mice by 96 hours (Fig. 8).

CBG-treated CLP-CBG mice exhibited a transient, marked reduction in plasma concentrations of IL-1β (36%), IL-6 (48%), IL-12 (44%), MIP-2 (58%), and TNFα (40%) at 12 hours relative to control CLP mice (all *P* ≤ .001), followed by an increase at 24 hours. IL-6 concentrations in CLP-CBG mice fell below those in control CLP mice at 48 and 96 hours (46% and 44%, respectively; *P* ≤ .001), whereas IL-1β, IL-12, MIP-2, and TNFα returned to concentrations observed in control CLP mice at these time points (all time effects *P* ≤ .001; Fig. 8). In stark contrast, IL-10 and IFN-β1 peaked higher in CLP-CBG mice at 12 hours than in control CLP mice (by 33% and 32%, respectively, both *P* ≤ .001) then despite a later decline, these anti-inflammatory cytokines remained markedly elevated in CLP-CBG mice to 96 hours relative to control CLP mice (46% and 87%, respectively, both *P* ≤ .001; Fig. 8F and 8G). Significant CBG treatment effects were observed for IL-1β (*P* = .048), IL-6 (*P* = .033), IL-10 (*P* ≤ .001), IL-12 (*P* = .037), and IFN-1β (*P* ≤ .001), all with time × treatment interactions (*P* ≤ .001), while IL-1β (71%), IL-6 (91%), IL-10 (96%), IL-12 (1.4-fold), TNFα (1.7-fold), and IFN-1β (18-fold) were higher in control CLP nonsurvivors compared to survivors (*P* ≤ .001 for all; Table 3).

#### Systemic damage markers

**Figure 9:**
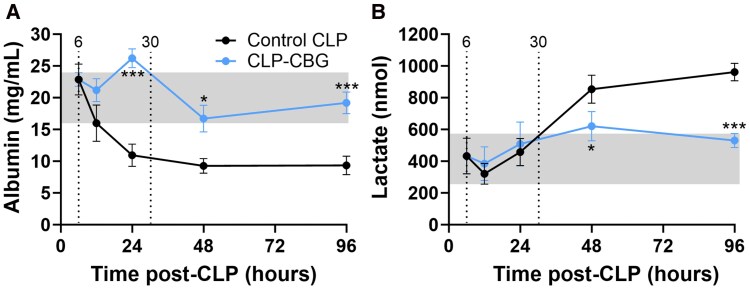
CBG therapy augments plasma albumin and lowers plasma lactate in CLP-sepsis. Time course series plasma profiles of (A) albumin and (B) lactate in control CLP and CLP-CBG mice (each n = 10 per timepoint) from 6 to 96 hours post-CLP. Vertical dotted lines mark times of vehicle (control CLP, black) or CBG administration (CLP-CBG, blue); Light gray region highlights the daily range of albumin or lactate concentration in age-matched, unoperated mice. Data are mean ± SEM; *P ≤ .05, **P ≤ .01, ***P ≤ .001 (CLP-CBG vs control CLP).

Plasma albumin and lactate profiles are illustrated in Fig. 9. Concentrations of both remained within their respective daily range in unoperated mice at 6 hours, prior to any intervention. Albumin concentrations then decreased in control CLP mice after 24 hours, with a 48-hour nadir (45%, *P* = .0032; time effect *P* ≤ .001; Fig. 9A), while lactate concentrations increased from 48 hours through to 96 hours (45%, *P* ≤ .001; time effect *P* ≤ .001; Fig. 9B). Conversely, CLP-CBG mice maintained albumin and lactate concentrations throughout the study period within their respective daily range in unoperated mice (CBG treatment effect, *P* = .003, *P* = .046, respectively; treatment × time effect, both *P* ≤ .001). Furthermore, control CLP nonsurvivors exhibited higher plasma lactate and lower albumin concentrations compared to survivors (both *P* ≤ .001; Table 3).

#### Organ damage markers

**Figure 10:**
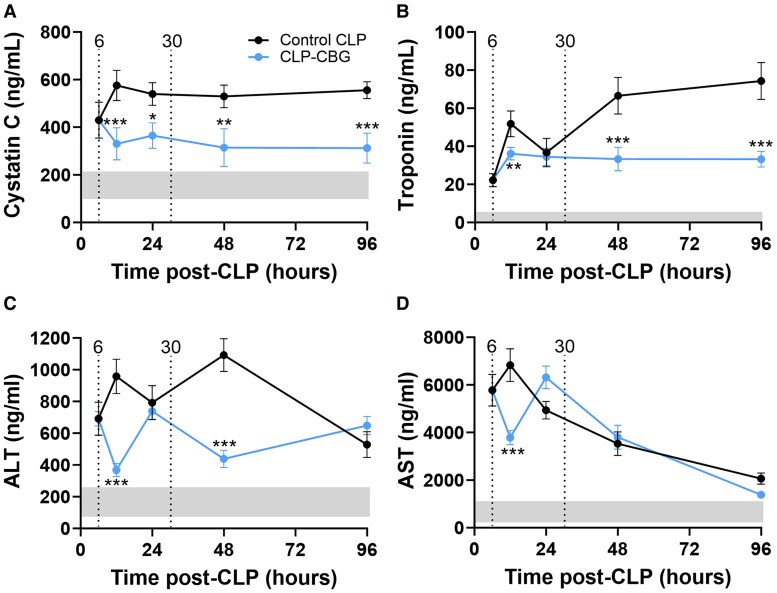
CBG therapy attenuates organ damage marked by plasma cystatin-C and troponin-I in CLP-sepsis. Time course series plasma profiles of (A) cystatin-C, (B) troponin-I, (C) alanine aminotransferase (ALT), and (D) aspartate aminotransferase (AST) in control CLP and CLP-CBG mice (each n = 10 per timepoint) from 6 to 96 hours post-CLP. Vertical dotted lines mark times of vehicle (control CLP, black) or CBG administration (CLP-CBG, blue); Light gray region highlights the daily range of cystatin-C, troponin-I, ALT, or AST concentration in age-matched, unoperated mice. Data are mean ± SEM; *P ≤ .05, **P ≤ .01, ***P ≤ .001 (CLP-CBG vs control CLP).

Plasma cystatin-C, troponin-I, ALT, and AST profiles are illustrated in Fig. 10. These increased from 6 hours, prior to any intervention, in control CLP mice (2.1-fold for cystatin-C, 6.3-fold for troponin-I, 1.7-fold for ALT, and 2.9-fold for AST; all *P* ≤ .001) and peaked at 12 hours (increases of 3.2-fold, 14.6-fold, 3.5-fold, and 2.8-fold, respectively; all *P* ≤ .001) relative to their daily range mean concentration in unoperated mice. Cystatin-C then remained elevated to 96 hours (2.8-fold, *P* ≤ .001; Fig. 10A), while troponin-I increased progressively to this time point in control CLP mice (50.7-fold, *P* ≤ .001; time effect *P* ≤ .001; Fig. 10B). ALT peaked at 48 hours with a 3.3-fold increase (*P* ≤ .001) before declining in control CLP mice, though remained elevated at 96 hours (1.3-fold, *P* = .045; time effect *P* = .005; Fig. 10C); AST concentrations returned to the AST daily range in unoperated mice by 96 hours (time effect *P* ≤ .001; Fig. 10D).

CBG therapy markedly attenuated the rise in plasma cystatin-C and troponin-I concentrations seen in control CLP in CLP-CBG mice, with lower cystatin-C evident beyond 12 hours and lower troponin-I at 12 hours and beyond 48 hours (CBG treatment effect, *P* ≤ .001 and *P* = .031, respectively, and a time × treatment effect, *P* ≤ .001, for both) with peak differences at 96 hours (43% and 55%; Fig. 10A and 10B). Plasma ALT concentrations in CLP-CBG mice also decreased transiently following CBG administration at 12 hours (59%, *P* ≤ .001) and 48 hours (60%, *P* ≤ .001), despite similar concentrations in control CLP and CLP-CBG mice at 24 and 96 hours (time effect *P* = .005; CBG treatment effect *P* ≤ .001, and a time × treatment interaction *P* ≤ .001; Fig. 10C). In contrast, plasma AST was transiently decreased only at 12 hours in CLP-CBG mice (43%, *P* = .002), then paralleled the response in control CLP mice (time effect *P* = .00 L, and a time × treatment interaction *P* = .042; Fig. 10D). Control CLP nonsurvivors exhibited higher plasma troponin-I (20%, *P* = .046) and AST (1.6-fold, *P* = .044) concentrations compared to survivors (Table 3).

## Discussion

Our study identifies plasma CBG depletion as a hallmark feature of high-grade CLP-sepsis in C57BL/6 mice, which parallels data from our observational study in humans (15, 18). This validates our precision model to investigate CBG replacement therapy. With this model, we demonstrate that intravenous CBG replacement therapy in CLP-CBG mice, sufficient to maintain plasma CBG within the daily range of healthy unoperated mice, yields marked mortality, morbidity, and physiological benefits, as well as comprehensive protection from inflammation, system, and organ damage. We also describe rapid, differential targeting of [^124^I]I-CBG to injured and inflamed tissues at the CLP injury and abdomen using PET imaging.

Our precision murine CLP model of high-grade sepsis revealed early, transient episodes of hypotension, with 60% of control CLP mice later progressing to septic shock, characterized by coincident hypotension and predefined hyperlactatemia (20% rise). Building on this, we also show rapid depletion of plasma CBG in control CLP mice from 6 hours, which coincided with hypotension, hypothermia, and the rapid emergence of an IL-6-driven systemic inflammatory response syndrome (SIRS). Plasma CBG concentrations below 7 µg/mL, typically beyond 48 hours, consistently signaled the onset of irreversible septic shock.

Our findings in control CLP mice align with our mechanistic clinical data that implicate CBG depletion below 200 nmol/L (167 mcg/dL) as the only potentially risk factor for a 3-fold increased risk of death in critically ill patients with septic shock (18). They also accord with previous research on male C57BL/6 mice deficient in plasma CBG due to hepatocyte-specific deletion of Kruppel-like factor-15 (KLF-15). These KLF-15-deficient mice, unable to transcribe *SERPINA6*, demonstrate impaired inflammatory responses and high mortality following lipopolysaccharide-induced acute sepsis (28). Other studies have demonstrated that CBG deficiency in humans is associated with hypotension (29-32), and that serum CBG concentration above 200nM reduces the requirement for vasopressor support in patients with septic shock (10, 20, 33). Together with current findings, this strongly positions CBG as a modulator of hemodynamic responses and mortality in sepsis.

CBG therapy in CLP-CBG mice limited weight loss, reduced morbidity (CDI), preserved normal plasma lactate and albumin concentrations, and comprehensively protected organs, evidenced by a halving of kidney (cystatin-C), cardiac (troponin-I), and liver transaminase (ALT) damage markers in CLP-CBG mice. CBG positively modulated the inflammatory response by decreasing pro-inflammatory cytokines IL-6 and the neutrophil attractant chemokine MIP-2, and mitigated excess total and free corticosterone, all while sustaining robust anti-inflammatory responses led by IL-10 and IFN-β1. CBG therapy reduced the total time in hypotension by three-quarters, more than halved the duration of the first hypotensive episode, and total time in hypothermia, and prevented progression to septic shock. Together, these comprehensive CBG benefits underpinned the marked reduction in relative CLP-sepsis mortality of over two-thirds, specifically, a profound reduction in CLP mortality from 58.3% to 16.6%. These broad-ranging benefits distinguish CBG therapy from the setbacks of previous immune-based sepsis treatments (34, 35) and provide a strong impetus to conduct a clinical trial to assess its potential for improving survival and recovery in human sepsis.

The sustained IL-10 and IFN-β1 increase in CLP-CBG mice is intuitively responsible for the attenuation in system and organ damage markers, particularly albumin, lactate, cystatin-C, and troponin-I; however, the precise roles of IL-10 and IFN-β1 in sepsis and septic shock remain enigmatic. Monoclonal IL-10 neutralization in C3H/HeN mice was shown to increase 7-day survival post-CLP, but only if administered after the initial pro-inflammatory hyperdynamic state at 12 hours (36), while IFN-β1-deficient C57BL/6 or IFN-β1-defective SPRET/Ei mice showed reduced mortality to lipopolysaccharide-induced sepsis (37, 38). In contrast, IL-10-deficient C57BL/6 mice and IFN-β1-deficient SEV129 mice showed increased CLP mortality compared to wild-type control mice (39). These studies differ in their genotypes and sepsis approaches but highlight the uncertainty on the precise mechanism of the early and sustained CBG-induced elevation in IL-10 and IFN-β1 in septic shock, which requires further research.

CBG administration at 6 hours also coincided with a marked, rapid, transient decline in pro-inflammatory cytokines, then a marked 12-hour rebound to peak concentrations beyond those in control CLP mice at 24 hours. Similar rebound phenomena have been reported on therapy-dependent lowering of pro-inflammatory cytokines across diverse inflammatory conditions (40), including multiple sclerosis (41), psoriasis (42), and rheumatoid arthritis (43), and is theorized to be due to homeostatic overcorrection (44-46). This rebound can be intensified by positive feedback loops within the inflammatory process (47-49); since CBG only masks the underlying cause of inflammation without treating the active infection, these loops are likely to quickly reactivate and amplify the pro-inflammatory response.

We noted that, despite evidence of severe systemic distress and circulatory failure framed by hyperlactatemia, hypothermia, and bradycardia (50, 51), the 2 nonsurviving CLP-CBG mice had normal blood pressure at early euthanasia, contrasting the profound hypotension of control CLP mice. This, together with clinical data in CBG-replete patients, adds support to the role of CBG in normalizing blood pressure in sepsis, whether through cortisol/corticosterone delivery or by attenuating pro-inflammatory cytokines, which are known to induce hypotension (52). This suggests that while CBG successfully prevents a drop in blood pressure, it does not always fully mitigate the underlying cellular and metabolic dysfunction that ultimately leads to mortality.

For the first time, PET imaging studies revealed the pharmacokinetic, pharmacodynamic, and molecular anatomy of [^124^I]I-CBG, showing generalized distribution and elimination profiles in healthy unoperated mice, but distinct and rapid targeting to the CLP-adjacent injury at the cecum-intestine and abdomen at 12 hours post-CLP. This targeting in CLP-CBG mice coincided with the lower SIRS peak, shorter hypotension and rapid decline in free corticosterone and plasma biomarkers of organ damage (cystatin-C, troponin-I, ALT) and adds fundamental insight that CBG therapy restores corticosterone carriage and targeting in an inflammation- and injury-dependent manner (13, 53, 54), to actively modulate SIRS. Lower mortality was associated with renal [^124^I]I-CBG uptake, and higher mortality with pancreatic [^124^I]I-CBG uptake. This may reflect positive actions of CBG on the kidneys, the major target organ in septic injury, while the negative association with pancreatic [^124^I]I-CBG uptake may arise due to pancreatitis, a known risk factor for increased mortality in patients with sepsis and septic shock (55, 56). A high [^124^I]I-CBG signal was noted in the stomach, which we attribute to physiological actions of the sodium iodide symporter (NIS) present in the gastric mucosa (57). NIS-dependent physiological [^124^I]I uptake is likely to also underpin this signal in the thyroid and salivary glands (58).

The acute septic state, driven by pro-inflammatory cytokines like TNF-α and IL-1β, induces 11β-HSD1 activity in immune cells, creating an intracellular mechanism to convert inactive 11-dehydrocorticosterone to active corticosterone, to amplify the local anti-inflammatory signal (59). We acknowledge that this in vivo response is complex (60), relying on a multifactorial network involving pituitary-adrenal regulation, the availability of NADPH for 11β-HSD1-mediated regeneration of active glucocorticoids, and the metabolic activity of mononuclear inflammatory cells. In this context, the decrease in both free and total corticosterone we observed with CBG therapy may be interpreted as an intrinsic anti-inflammatory response to CBG, as reflected in the altered cytokine profile. Hence, the survival benefit of CBG, which exceeds that of glucocorticoids in septic shock (61), may be due to better targeted corticosterone delivery to the inflammatory site, as shown by our PET imaging, and/or a possible direct immunomodulatory effect of CBG. The existence of such immunomodulatory effects, and whether they reflect better targeted corticosterone delivery or other immune effects, require further immunological study.

However, reliance on corticosterone delivery alone may be nullified in the 60% of sepsis–septic shock patients with glucocorticoid resistance (62); such resistance is also seen in C57BL/6 mice following CLP (61). The acute effects of corticosterone are stimulatory on IL-10 and inhibitory on IFN-1β (60, 63, 64); CBG lowered corticosterone, but the magnitude of the effect on IL-10 and the paradoxical rise in IFN-1β adds strong support that the novel cytokine effects described are intrinsic to CBG rather than corticosterone-mediated. This unique immune action of CBG may explain its efficacy, contrasting the ineffectiveness of adjunctive glucocorticoid therapy in murine CLP-sepsis and clinical septic shock (65). These findings underscore the critical role of CBG in the stress response to sepsis, where CBG depletion may impair a vital anti-inflammatory “circuit breaker” to worsen organ damage. Indeed, serpin family protease inhibitors are crucial for limiting inflammation and preventing tissue damage; Serpin B1 inhibits the formation of neutrophil extracellular traps (66), while Serpin A3 targets cathepsin G and mast cell chymotrypsin, both key mediators of inflammatory tissue destruction (67). Although CBG lacks protease activity, its complex tertiary structure and glycobiology suggest diverse interactions with downstream signaling partners or pathways, the repertoire and nature of which require further elucidation.

We acknowledge limitations in study design. We first administered CBG therapy at 6 hours, timed to standardized early sequelae of hypotension in CLP mice, a feature that preceded the observed 12-hour SIRS peak. We acknowledge that this report deals with induced septic shock, whereas clinical protocols initiate treatment immediately upon sepsis diagnosis to prevent shock. Furthermore, clinical presentations are often delayed, making it challenging to ascertain sepsis onset (68), and are heterogeneous regarding infection site, and immune status (69, 70). However, our human studies indicate that CBG depletion in sepsis is less severe than in septic shock (10). Given that 18% to 23% of patients admitted with sepsis deteriorate to septic shock within 72 hours (71), this frames a therapeutic window wherein deployment of CBG therapy as an adjunct to standard care in CBG-deplete patients offers a critical opportunity to prevent this progression. We also acknowledge that CBG therapy does not mimic the daily range of CBG concentrations in unoperated C57BL/6 mice, and that study findings were limited to male mice, chosen to avoid the confounding of the estrous cycle stage on corticosterone (72) at proof-of-concept stage. Follow-up research is needed to extend these investigations to female C57BL/6 mice and to systematically map CBG therapy outcomes across estrous cycle stages.

## Conclusion

Building on our seminal clinical findings of the strong association between CBG depletion and mortality in sepsis, this preclinical study has utilized our refined murine model of polymicrobial sepsis with telemetry and precision plasma biomarkers to demonstrate profound benefits of CBG therapy across hemodynamic, systemic, organ, morbidity, and mortality domains. These results provide a powerful impetus to evaluate recombinant CBG replacement therapy in patients with sepsis and septic shock, as a novel therapy with the potential to fundamentally remap the disease course.

## Data Availability

Restrictions apply to the availability of some, or all data generated or analyzed during this study to preserve patient confidentiality or because they were used under license. The corresponding author will on request detail the restrictions and any conditions under which access to some data may be provided.

## Abbreviations

ALT: alanine aminotransferase
AST: aspartate aminotransferase
CBG: corticosteroid-binding globulin
CDI: cumulative disease index
CLP: cecal ligation and puncture
ELISA: enzyme-linked immunosorbent assay
ICU: intensive care unit
IFN-β1: interferon-β1
IL-: interleukin
iTLC: instant thin-layer chromatography
IVC: individually ventilated cage
MAP: mean arterial pressure
MIP-2: macrophage inflammatory protein 2
PBS: phosphate-buffered saline
PBS-T: phosphate-buffered saline with Tween 20
PET: positron emission tomography
SIRS: systemic inflammatory response syndrome
SUV: standardized uptake value
TNFα: tumor necrosis factor α
VOI: volume of interest

## References

[1] Srzic I, Nesek Adam V, Tunjic Pejak D. Sepsis definition: what's new in the treatment guidelines. Acta Clin Croat. 2022;61(Suppl 1):67‐72. 10.20471/acc.2022.61.s1.11 PMC9536156 36304809

[2] Rudd KE, Johnson SC, Agesa KM, et al Global, regional, and national sepsis incidence and mortality, 1990-2017: analysis for the global burden of disease study. Lancet. 2020;395(10219):200‐211. 31954465 10.1016/S0140-6736(19)32989-7 PMC6970225

[3] Finfer S, Bellomo R, Lipman J, French C, Dobb G, Myburgh J. Adult-population incidence of severe sepsis in Australian and New Zealand intensive care units. Intensive Care Med. 2004;30(4):589‐596. 14963646 10.1007/s00134-004-2157-0

[4] Finfer S, Glass P, Todorovski V, et al Stopping Sepsis: A National Action Plan. George Institute for Global Health; 2017.

[5] Paoli CJ, Reynolds MA, Sinha M, Gitlin M, Crouser E. Epidemiology and costs of sepsis in the United States—an analysis based on timing of diagnosis and severity level. Crit Care Med. 2018;46(12):1889‐1897. 30048332 10.1097/CCM.0000000000003342 PMC6250243

[6] Arefian H, Heublein S, Scherag A, et al Hospital-related cost of sepsis: a systematic review. J Infect. 2017;74(2):107‐117. 27884733 10.1016/j.jinf.2016.11.006

[7] Bauer M, Gerlach H, Vogelmann T, Preissing F, Stiefel J, Adam D. Mortality in sepsis and septic shock in Europe, North America and Australia between 2009 and 2019-results from a systematic review and meta-analysis. Crit Care. 2020;24(1):239. 32430052 10.1186/s13054-020-02950-2 PMC7236499

[8] Evans T . Diagnosis and management of sepsis. Clin Med (Lond). 2018;18(2):146‐149. 29626019 10.7861/clinmedicine.18-2-146 PMC6303466

[9] Coopersmith CM, De Backer D, Deutschman CS, et al Surviving Sepsis Campaign: research priorities for sepsis and septic shock. Crit Care Med. 2018;46(8):1334‐1356. 29957716 10.1097/CCM.0000000000003225

[10] Meyer EJ, Nenke MA, Rankin W, et al Total and high-affinity corticosteroid-binding globulin depletion in septic shock is associated with mortality. Clin Endocrinol (Oxf). 2019;90(1):232‐240. 30160799 10.1111/cen.13844

[11] Gardill BR, Vogl MR, Lin HY, Hammond GL, Muller YA. Corticosteroid-binding globulin: structure-function implications from species differences. PLoS One. 2012;7(12):e52759. 23300763 10.1371/journal.pone.0052759 PMC3530532

[12] Chan WL, Carrell RW, Zhou A, Read RJ. How changes in affinity of corticosteroid-binding globulin modulate free cortisol concentration. J Clin Endocrinol Metab. 2013;98(8):3315‐3322. 23783094 10.1210/jc.2012-4280 PMC3813945

[13] Nenke MA, Nielsen ST, Lehrskov LL, et al Pyrexia's effect on the CBG-cortisol thermocouple, rather than CBG cleavage, elevates the acute free cortisol response to TNF-alpha in humans. Stress. 2017;20(2):183‐188. 28166688 10.1080/10253890.2017.1292420

[14] Sumer-Bayraktar Z, Grant OC, Venkatakrishnan V, Woods RJ, Packer NH, Thaysen-Andersen M. Asn347 glycosylation of corticosteroid-binding globulin fine-tunes the host immune response by modulating proteolysis by Pseudomonas aeruginosa and neutrophil elastase. J Biol Chem. 2016;291(34):17727‐17742. 27339896 10.1074/jbc.M116.735258 PMC5016167

[15] Nyberg L, Marekov LN, Jones I, Lundquist G, Jornvall H. Characterization of the murine corticosteroid binding globulin: variations between mammalian forms. J Steroid Biochem. 1990;35(1):61‐65. 2407901 10.1016/0022-4731(90)90146-j

[16] Lee JH, Meyer EJ, Nenke MA, Falhammar H, Torpy DJ. Corticosteroid-binding globulin (CBG): spatiotemporal distribution of cortisol in sepsis. Trends Endocrinol Metab. 2023;34(3):181‐190. 36681594 10.1016/j.tem.2023.01.002

[17] Bartalena L, Hammond GL, Farsetti A, Flink IL, Robbins J. Interleukin-6 inhibits corticosteroid-binding globulin synthesis by human hepatoblastoma-derived (hep G2) cells. Endocrinology. 1993;133(1):291‐296. 8391424 10.1210/endo.133.1.8391424

[18] Sam S, Corbridge TC, Mokhlesi B, Comellas AP, Molitch ME. Cortisol levels and mortality in severe sepsis. Clin Endocrinol (Oxf). 2004;60(1):29‐35. 14678284 10.1111/j.1365-2265.2004.01923.x

[19] Ho JT, Al-Musalhi H, Chapman MJ, et al Septic shock and sepsis: a comparison of total and free plasma cortisol levels. J Clin Endocrinol Metab. 2006;91(1):105‐114. 16263835 10.1210/jc.2005-0265

[20] Meyer EJ, Nenke MA, Davies ML, et al Corticosteroid-binding globulin deficiency independently predicts mortality in septic shock. J Clin Endocrinol Metab. 2022;107(6):1636‐1646. 35152290 10.1210/clinem/dgac035

[21] Percie du Sert N, Hurst V, Ahluwalia A, et al The ARRIVE guidelines 2.0: updated guidelines for reporting animal research. BMJ Open Sci. 2020;4(1):e100115. 10.1136/bmjos-2020-100115 PMC7610906 34095516

[22] Otero-Anton E, Gonzalez-Quintela A, Lopez-Soto A, Lopez-Ben S, Llovo J, Perez LF. Cecal ligation and puncture as a model of sepsis in the rat: influence of the puncture size on mortality, bacteremia, endotoxemia and tumor necrosis factor alpha levels. Eur Surg Res. 2001;33(2):77‐79. 11399872 10.1159/000049698

[23] Ramsay S, Kilgariff DE, Young BJ, et al 2025. Supplementary methods and data for Harnessing a native corticosteroid-binding globulin to treat life-threatening septic shock. Dataset. 10.25909/30560000.v3. PMC12848944 41521757

[24] Cuenca AG, Delano MJ, Kelly-Scumpia KM, Moldawer LL, Efron PA. Cecal ligation and puncture. Curr Protoc Immunol. 2010;91(1). Chapter 19:Unit 19.13. 10.1002/0471142735.im1913s91 PMC3058382 21053304

[25] Hill A, Khalil H, Laborc K, et al Corticosteroid treatment during sepsis alters hippocampal function in male and female survivors. Biol Psychiatry Glob Open Sci. 2024;4(1):336‐345. 38298779 10.1016/j.bpsgos.2023.08.001 PMC10829652

[26] Coolens JL, Van Baelen H, Heyns W. Clinical use of unbound plasma cortisol as calculated from total cortisol and corticosteroid-binding globulin. J Steroid Biochem. 1987;26(2):197‐202. 3560936 10.1016/0022-4731(87)90071-9

[27] Lauridsen MD, Gammelager H, Schmidt M, Nielsen H, Christiansen CF. Positive predictive value of international classification of diseases, 10th revision, diagnosis codes for cardiogenic, hypovolemic, and septic shock in the Danish National Patient Registry. BMC Med Res Methodol. 2015;15(1):23. 25888061 10.1186/s12874-015-0013-2 PMC4373092

[28] Jiang Z, Elsarrag SZ, Duan Q, et al KLF15 cistromes reveal a hepatocyte pathway governing plasma corticosteroid transport and systemic inflammation. Sci Adv. 2022;8(10):eabj2917. 35263131 10.1126/sciadv.abj2917 PMC8906731

[29] Torpy DJ, Bachmann AW, Gartside M, et al Association between chronic fatigue syndrome and the corticosteroid-binding globulin gene ALA SER224 polymorphism. Endocr Res. 2004;30(3):417‐429. 15554358 10.1081/erc-200035599

[30] Torpy DJ, Bachmann AW, Grice JE, et al Familial corticosteroid-binding globulin deficiency due to a novel null mutation: association with fatigue and relative hypotension. J Clin Endocrinol Metab. 2001;86(8):3692‐3700. 11502797 10.1210/jcem.86.8.7724

[31] Torpy DJ, Ho JT. Corticosteroid-binding globulin gene polymorphisms: clinical implications and links to idiopathic chronic fatigue disorders. Clin Endocrinol (Oxf). 2007;67(2):161‐167. 17547679 10.1111/j.1365-2265.2007.02890.x

[32] Gagliardi L, Ho JT, Torpy DJ. Corticosteroid-binding globulin: the clinical significance of altered levels and heritable mutations. Mol Cell Endocrinol. 2010;316(1):24‐34. 19643166 10.1016/j.mce.2009.07.015

[33] Nenke MA, Rankin W, Chapman MJ, et al Depletion of high-affinity corticosteroid-binding globulin corresponds to illness severity in sepsis and septic shock; clinical implications. Clin Endocrinol (Oxf). 2015;82(6):801‐807. 25409953 10.1111/cen.12680

[34] Jain A, Singam A, Mudiganti VNKS. Recent advances in immunomodulatory therapy in sepsis: a comprehensive review. Cureus. 2024;16(3):e57309. 38690455 10.7759/cureus.57309 PMC11059166

[35] Wu Y, Wang L, Li Y, et al Immunotherapy in the context of sepsis-induced immunological dysregulation. Front Immunol. 2024;15:1391395. 38835773 10.3389/fimmu.2024.1391395 PMC11148279

[36] Song GY, Chung CS, Chaudry IH, Ayala A. What is the role of interleukin 10 in polymicrobial sepsis: anti-inflammatory agent or immunosuppressant? Surgery. 1999;126(2):378‐383. 10455909

[37] Mahieu T, Park JM, Revets H, et al The wild-derived inbred mouse strain SPRET/Ei is resistant to LPS and defective in IFN-beta production. Proc Natl Acad Sci U S A. 2006;103(7):2292‐2297. 16455798 10.1073/pnas.0510874103 PMC1413734

[38] Karaghiosoff M, Steinborn R, Kovarik P, et al Central role for type I interferons and Tyk2 in lipopolysaccharide-induced endotoxin shock. Nat Immunol. 2003;4(5):471‐477. 12679810 10.1038/ni910

[39] Latifi SQ, O'Riordan MA, Levine AD. Interleukin-10 controls the onset of irreversible septic shock. Infect Immun. 2002;70(8):4441‐4446. 12117955 10.1128/IAI.70.8.4441-4446.2002 PMC128185

[40] Gatti M, Pea F. The cytokine release syndrome and/or the proinflammatory cytokines as underlying mechanisms of downregulation of drug metabolism and drug transport: a systematic review of the clinical pharmacokinetics of victim drugs of this drug-disease interaction under different clinical conditions. Clin Pharmacokinet. 2022;61(11):1519‐1544. 36059001 10.1007/s40262-022-01173-8 PMC9441320

[41] Juto A, Fink K, Al Nimer F, Piehl F. Interrupting rituximab treatment in relapsing-remitting multiple sclerosis; no evidence of rebound disease activity. Mult Scler Relat Disord. 2020;37:101468. 31683231 10.1016/j.msard.2019.101468

[42] Harrold LR, Stolshek BS, Rebello S, et al Rebound in measures of disease activity and symptoms in Corrona Registry patients with psoriatic arthritis who discontinue tumor necrosis factor inhibitor therapy after achieving low disease activity. J Rheumatol. 2018;45(1):78‐82. 28966209 10.3899/jrheum.161567

[43] Teixeira MZ . Rebound effect of modern drugs: serious adverse event unknown by health professionals. Rev Assoc Med Bras (1992). 2013;59(6):629‐638. 24211013 10.1016/j.ramb.2013.05.003

[44] Teixeira MZ . Immunomodulatory drugs (natalizumab), worsening of multiple sclerosis, rebound effect and similitude. Homeopathy. 2013;102(3):215‐224. 23870382 10.1016/j.homp.2013.05.001

[45] Teixeira MZ . Biological therapies (immunomodulatory drugs), worsening of psoriasis and rebound effect: new evidence of similitude. Homeopathy. 2016;105(4):344‐355. 27914574 10.1016/j.homp.2016.09.002

[46] Aston PJ, Derks G, Agoram BM, van der Graaf PH. A mathematical analysis of rebound in a target-mediated drug disposition model: II. With feedback. J Math Biol. 2017;75(1):33‐84. 27832321 10.1007/s00285-016-1073-6 PMC5487209

[47] Yun AJ, Lee PY, Bazar KA. Paradoxical strategy for treating chronic diseases where the therapeutic effect is derived from compensatory response rather than drug effect. Med Hypotheses. 2005;64(5):1050‐1059. 15780510 10.1016/j.mehy.2004.09.007

[48] Rozengurt E, Soares HP, Sinnet-Smith J. Suppression of feedback loops mediated by PI3K/mTOR induces multiple overactivation of compensatory pathways: an unintended consequence leading to drug resistance. Mol Cancer Ther. 2014;13(11):2477‐2488. 25323681 10.1158/1535-7163.MCT-14-0330 PMC4222988

[49] Altintas DM, Cerqua M, De Laurentiis A, et al An mTOR feedback loop mediates the ‘flare’ (‘rebound’) response to MET tyrosine kinase inhibition. Sci Rep. 2023;13(1):1378. 36697438 10.1038/s41598-023-28648-3 PMC9876934

[50] Secher NH, Bie P. Bradycardia during reversible haemorrhagic shock-a forgotten observation? Clin Physiol. 1985;5(4):315‐323. 3899474 10.1111/j.1475-097x.1985.tb00752.x

[51] Schlotman TE, Suresh MR, Koons NJ, et al Predictors of hemodynamic decompensation in progressive hypovolemia: compensatory reserve versus heart rate variability. J Trauma Acute Care Surg. 2020;89(Suppl 2):S161‐S168. 32044875 10.1097/TA.0000000000002605

[52] Periasamy S, Chu PY, Li YH, Hsu DZ, Liu MY. Sesamol ameliorates hypotension by modulating cytokines and PPAR-gamma in systemic inflammatory response. EXCLI J. 2015;14:948‐957. 26839527 10.17179/excli2015-367 PMC4732502

[53] Lee JH, Meyer EJ, Nenke MA, Lightman SL, Torpy DJ. Cortisol, stress, and disease—bidirectional associations; role for corticosteroid-binding globulin? J Clin Endocrinol Metab. 2024;109(9):2161‐2172. 38941154 10.1210/clinem/dgae412

[54] Meyer EJ, Torpy DJ, Chernykh A, et al Pyrexia and acidosis act independently of neutrophil elastase reactive center loop cleavage to effect cortisol release from corticosteroid-binding globulin. Protein Sci. 2020;29(12):2495‐2509. 33085168 10.1002/pro.3982 PMC7679970

[55] Chaari A, Abdel Hakim K, Bousselmi K, et al Pancreatic injury in patients with septic shock: a literature review. World J Gastrointest Oncol. 2016;8(7):526‐531. 27559431 10.4251/wjgo.v8.i7.526 PMC4942740

[56] Teblick A, Gunst J, Van den Berghe G. Critical illness-induced corticosteroid insufficiency: what it is not and what it could be. J Clin Endocrinol Metab. 2022;107(7):2057‐2064. 35358303 10.1210/clinem/dgac201 PMC9202732

[57] Ravera S, Reyna-Neyra A, Ferrandino G, Amzel LM, Carrasco N. The sodium/iodide symporter (NIS): molecular physiology and preclinical and clinical applications. Annu Rev Physiol. 2017;79:261‐289. 28192058 10.1146/annurev-physiol-022516-034125 PMC5739519

[58] Pesce L, Kopp P. Iodide transport: implications for health and disease. Int J Pediatr Endocrinol. 2014;2014(1):8. 25009573 10.1186/1687-9856-2014-8 PMC4089555

[59] Esteves CL, Verma M, Rog-Zielinska E, et al Pro-inflammatory cytokine induction of 11beta-hydroxysteroid dehydrogenase type 1 in A549 cells requires phosphorylation of C/EBPbeta at Thr235. PLoS One. 2013;8(9):e75874. 24086653 10.1371/journal.pone.0075874 PMC3784397

[60] Koldzic-Zivanovic N, Tu H, Juelich TL, et al Regulation of adrenal glucocorticoid synthesis by interleukin-10: a preponderance of IL-10 receptor in the adrenal zona fasciculata. Brain Behav Immun. 2006;20(5):460‐468. 16256304 10.1016/j.bbi.2005.09.003

[61] Vandewalle J, Timmermans S, Paakinaho V, et al Combined glucocorticoid resistance and hyperlactatemia contributes to lethal shock in sepsis. Cell Metab. 2021;33(9):1763‐1776.e5. 34302744 10.1016/j.cmet.2021.07.002

[62] Dendoncker K, Libert C. Glucocorticoid resistance as a major drive in sepsis pathology. Cytokine Growth Factor Rev. 2017;35:85‐96. 28479044 10.1016/j.cytogfr.2017.04.002

[63] Qin Z, Shi DD, Li W, et al Berberine ameliorates depression-like behaviors in mice via inhibiting NLRP3 inflammasome-mediated neuroinflammation and preventing neuroplasticity disruption. J Neuroinflammation. 2023;20(1):54. 36859349 10.1186/s12974-023-02744-7 PMC9976521

[64] Jalkanen J, Pettila V, Huttunen T, Hollmen M, Jalkanen S. Glucocorticoids inhibit type I IFN beta signaling and the upregulation of CD73 in human lung. Intensive Care Med. 2020;46(10):1937‐1940. 32430515 10.1007/s00134-020-06086-3 PMC7235433

[65] Venkatesh B, Finfer S, Cohen J, et al Adjunctive glucocorticoid therapy in patients with septic shock. N Engl J Med. 2018;378(9):797‐808. 29347874 10.1056/NEJMoa1705835

[66] Torriglia A, Martin E, Jaadane I. The hidden side of SERPINB1/leukocyte elastase inhibitor. Semin Cell Dev Biol. 2017;62:178‐186. 27422329 10.1016/j.semcdb.2016.07.010 PMC5610702

[67] Soman A, Asha Nair S. Unfolding the cascade of SERPINA3: inflammation to cancer. Biochim Biophys Acta Rev Cancer. 2022;1877(5):188760. 35843512 10.1016/j.bbcan.2022.188760

[68] Leisman DE, Angel C, Schneider SM, D'Amore JA, D'Angelo JK, Doerfler ME. Sepsis presenting in hospitals versus emergency departments: demographic, resuscitation, and outcome patterns in a multicenter retrospective cohort. J Hosp Med. 2019;14(6):340‐348. 30986182 10.12788/jhm.3188 PMC6625440

[69] Barnato AE, Alexander SL, Linde-Zwirble WT, Angus DC. Racial variation in the incidence, care, and outcomes of severe sepsis: analysis of population, patient, and hospital characteristics. Am J Respir Crit Care Med. 2008;177(3):279‐284. 17975201 10.1164/rccm.200703-480OC PMC2720103

[70] Arina P, Hofmaenner DA, Singer M. Definition and epidemiology of sepsis. Semin Respir Crit Care Med. 2024;45(4):461‐468. 38968960 10.1055/s-0044-1787990

[71] Glickman SW, Cairns CB, Otero RM, et al Disease progression in hemodynamically stable patients presenting to the emergency department with sepsis. Acad Emerg Med. 2010;17(4):383‐390. 20370777 10.1111/j.1553-2712.2010.00664.x PMC4283798

[72] Aoki M, Shimozuru M, Kikusui T, Takeuchi Y, Mori Y. Sex differences in behavioral and corticosterone responses to mild stressors in ICR mice are altered by ovariectomy in peripubertal period. Zoolog Sci. 2010;27(10):783‐789. 20887175 10.2108/zsj.27.783

